# The enhancement of plant secondary metabolites content in *Lactuca sativa* L. by encapsulated bioactive agents

**DOI:** 10.1038/s41598-020-60690-3

**Published:** 2020-02-28

**Authors:** Slaven Jurić, Katarina Sopko Stracenski, Żaneta Król-Kilińska, Ivanka Žutić, Sanja Fabek Uher, Edyta Đermić, Snježana Topolovec-Pintarić, Marko Vinceković

**Affiliations:** 1 University of Zagreb, Faculty of Agriculture, Department of Chemistry, Zagreb, Croatia; 2 Wrocław University of Environmental and Life Sciences, Faculty of Biotechnology and Food Sciences, Department of Functional Food Products Development, Wrocław, Poland; 3 University of Zagreb, Faculty of Agriculture, Department of Vegetable Crops, Zagreb, Croatia; 4 University of Zagreb, Faculty of Agriculture, Department of Plant Pathology, Zagreb, Croatia

**Keywords:** Field trials, Environmental impact, Sustainability

## Abstract

Encapsulated bioactive agents applied to the *Lactuca sativa* L. present an innovative approach to stimulate the production of plant secondary metabolites increasing its nutritive value. Calcium and copper ions were encapsulated in biopolymeric microparticles (microspheres and microcapsules) either as single agents or in combination with biocontrol agents, *Trichoderma viride* spores, a fungal plant growth mediator. Both, calcium and copper ions are directly involved in the synthesis of plant secondary metabolites and alongside, *Trichoderma viride* can provide indirect stimulation and higher uptake of nutrients. All treatments with microparticles had a positive effect on the enhancement of plant secondary metabolites content in *Lactuca sativa* L. The highest increase of chlorophylls, antioxidant activity and phenolic was obtained by calcium-based microparticles in both, conventionally and hydroponically grown lettuces. Non-encapsulated fungus *Trichoderma viride* enhanced the synthesis of plant secondary metabolites only in hydroponics cultivation signifying the importance of its encapsulation. Encapsulation proved to be simple, sustainable and environmentally favorable for the production of lettuce with increased nutritional quality, which is lettuce fortified with important bioactive compounds.

## Introduction

Plant secondary metabolites (PSM) are natural sources of biologically active compounds used for a healthy diet, in traditional medicine and in a wide range of industrial applications^1^. The interest in enhancing PSM production is focused to obtain high yields suitable for commercial exploitation. Plant content of secondary plant metabolites is affected by genetic, environmental, and agronomic factors^2^. A variety of strategies (screening and selection of high-yielding cell lines, the culture of cells from various plant parts, suspension culture, induction by elicitors, metabolic engineering, optimizing media, plant growth regulators, *etc*.)^3^ as well as treatments with microspheres loaded with chemical and biological agents^4^ were used for enhancing PSM production in plant cell culture.

PSM such as polyphenols encompasses several classes of structurally diverse natural products biogenetically arising from the shikimate-phenylpropanoids-flavonoids pathways. Plants require these compounds for pigmentation, growth, reproduction, resistance to pathogens and for many other functions and they represent the adaptive characteristics that were subjected to the natural selection during evolution. In comparison to the animals, plants synthesize a broader spectrum of PSM because of the immobility and impossibility to escape predators, thus they evolved such a chemically based defense against predators^5^. The number of plant secondary metabolites in fresh lettuce can be improved with the addition of desirable compounds during the growth which is readily available for the plant root uptake. Higher PSM share would also have an important impact on human health by improving the antioxidant and nutrient intake through the human diet^6,7^.

With the broad spectrum of different secondary metabolites, plants can respond to diverse enemies and stressors. Since the production of the specific resistance traits can be extremely costly, new ways of defense enhancements should be employed. Methods involving increasing the expression of endogenous compounds could greatly influence plant resistance characteristics against plant attackers^5^.

Living microorganisms can be applied to the seeds, plant surfaces, or in the soil, in order to colonize the rhizosphere or the interior of the plant and promote growth by increasing the supply or availability of primary nutrients to the host plant^8^. Inoculation with Arbuscular mycorrhizal fungi enhances phenolics content and increases the antioxidant activity of lettuce leaves^9^, but efficient formulation demands a carrier material for living microorganism which must keep its functional properties after application.

One of the ways for site-specific delivery of living microorganisms is their encapsulation. Encapsulation is always developing technology that is superior to the other formulations in terms of living microorganisms protection from the harsh environment, with improved viability and the possibility of controlled and targeted release into the field^10^. Studies showed that *Trichoderma* species may induce changes in the microbiota composition of roots, enhance nutrient uptake, stabilize soil nutrients, promote root development, and increase root hair formation^11^. The dual roles of antagonistic activity against plant pathogens and the promotion of soil fertility make *Trichoderma* species a promising alternative to standard plant protection and nutrition methods.

Calcium ions are an essential part that plays an important role in the structure and permeability of cell membranes, plant cell division and elongation, carbohydrate translocation and N-metabolism^12,13^. Calcium cations also play a regulatory role in signal transduction and in the absorption of nutrients across the cell membranes^13–15^. Ca^2+^ has a role in signaling and helps in the upregulation of respective genes for polyphenols biosynthesis^16^. Ca^2+^ binds to the membrane phospholipids thus stabilizing the lipid bilayer and providing the structural integrity^17,18^ and is exhibited by the reduced malondialdehyde content in the plants treated with Ca^2+^ ^14,19,20^. Ca^2+^ is generally found in soil but it is relatively insoluble (*e.g*. CaCO_3_) in prevalent form. *Trichoderma* species acidify their surrounding environment by secreting organic acids and are able to solubilize phosphates, micronutrients and mineral cations^21^. From the other side, the simultaneous addition of calcium cations together with biocontrol agents improves the activity of biocontrol agents, that is, through a synergistic act^22^.

Copper ions show a stimulatory effect on the production of secondary metabolites in plants. They can induce synthesis of PSM with a positive effect on alkaloid production, synthesis of shikonin^23,24^, the production of digitalin^25^ and betalains^26^. Even though Cu^2+^ is a micro-constituent of growth media and is known to be essential for several biochemical and physiological pathways^27^ at higher concentrations it becomes toxic^28^. Therefore it is important to control the dosage of copper ions over the plant maturation time and to minimize the release into the environment which can be achieved by encapsulation.

Encapsulation results with more efficient use of chemicals and a convenient way of nutrients delivery for ecological and sustainable plant production^29–33^. Optimization of the encapsulation process is important to obtain microparticles with desirable traits. In our previous work, we have prepared microparticles for further applications^29–33^. This research introduces the application of optimized microparticles for the strategic delivery of active compounds to the plant (in this case lettuce) throughout the whole period of maturation. Not only with the intention to increase PSM to repel predators and pathogens but, consequently, also to obtain functional foods, lettuce fortified with important bioactive compounds.

## Materials and Methods

### Materials

Low-viscosity sodium alginate (CAS Registry No. 9005-38-3; A1112, Brookfield viscosity 4−12 cPs (1% in H_2_O at 25 °C)) and low molecular weight chitosan (CAS Registry No. 9012-76-4; 448869, molecular weight 100,000−300,000) were purchased from Sigma Aldrich (USA). All other chemicals were of analytical grade and used as received without further purification.

An indigenous isolate of *T. viride* originated from parasitized sclerotia of *Sclerotinia sclerotiorum* was used in all experiments^34^. To obtain spore suspensions, the fungus *Trichoderma viride* was grown in potato dextrose broth. Preparation of *T. viride* suspension was previously described^29^. Supplementary Fig. S1 presents macrophotograph of *T. viride* growing in a Petri dish (a), and microphotographs of *T. viride* mycelium (b) and spores suspension (c) taken under CLSM microscope^29,30^.

### Preparation of microparticles, application in the field and growth conditions

A two-year research (2017 and 2018) on the ground field (conventional cultivation - CC) and a parallel one year research (2018) in the hydroponic type of cultivation (HC) of green lettuce (*Lactuca sativa* L. var. crispa cv. ‘Melina’) have been investigated with regards to the application of microparticles loaded with different active compounds. Our preliminary trial (2017) revealed no significant influence on the morphology of treated lettuces but significant influences on the chlorophyll’s content. The same procedure was repeated in 2018 and additionally, parallel research in hydroponics was performed to observe influence in two different environments under different conditions.

**Figure 1:**
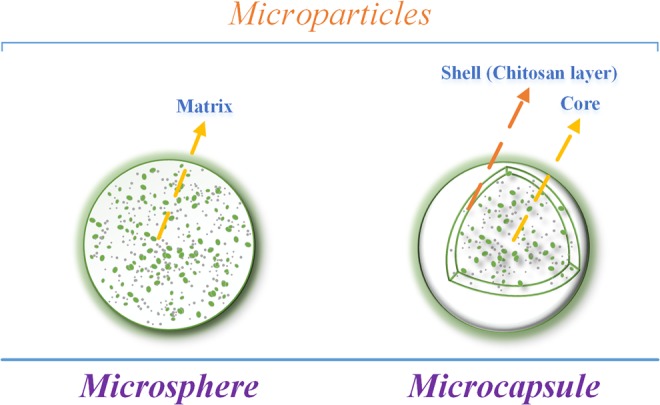
Schematic presentation of a microsphere and a microcapsule.

In accordance with our previous research^29–33^ after the optimization, eight different types of microparticles (microspheres and microcapsules) have been prepared and used in this research. Microspheres (Fig. 1) were prepared by dripping 1.5% sodium alginate (carrier) or a mixture of sodium alginate and *T. viride* spores into a cross-linking solution of 1% CaCl_2_ or CuSO_4_ × 5H_2_O. The production of microspheres was achieved with Encapsulator (Büchi-B390, BÜCHI Labortechnik AG, Switzerland) with the flow rate of carrier solution of 30 to 40 mL min^−1^ (determined by using encapsulator nozzle size of 1000 μm) at the vibration frequency of 40 Hz and the pressure of 0.3 bar. Microspheres were formed in the cross-linking solution under mechanical stirring, then washed several times with distilled water and filtered through Büchner funnel.

Microcapsules (Fig. 1) were prepared by dispersing microspheres in chitosan solution (0.5% chitosan in 1.0% CH_3_COOH) under constant stirring for 30 minutes. Obtained microcapsules were filtered, washed with distilled water and saline buffer. Microparticles were used the next day in the field. Suspension of *T. viride* spores in saline solution (0.85%) was used as side control (non-encapsulated), with control as non-treated samples. Two different types of cultivation were used, conventional in soil and in hydroponics, in order to compare the differences between cultivation type and to observe the influence on the same sample in different types of cultivation. Alginate-based microspheres containing either only chemical (Ca^2+^ or Cu^2+^) or both chemical and biological agents (*T. viride* spores - *Tv*) and microcapsules containing the aforementioned but with the addition of chitosan layer (suffix *-c*) are labeled and treatment samples are listed in Table 1.

**Table 1:** Treatment labels for conventional and hydroponic cultivation, used chemical agents (gelling cations), presence of biological agent (*T. viride*) in microparticles.

Treatment labels (Conventional/Hydroponics)	Chitosan coating	Gelling cation/ chemical agent	Chemical agent share in 4 g of microparticles	Biocontrol agent (*T. viride*)	Number of *T. viride* spores in 4 g of microparticles
*Ca*	−	Ca^2+^	9.87 ± 0.41 mg	−	−
*H-Ca*					
*Cu*	−	Cu^2+^	8.44 ± 0.24 mg	−	−
*H-Cu*					
*Ca/Tv*	−	Ca^2+^	10.08 ± 0.65 mg	+	8.8 × 10^4^
*H-Ca/Tv*					
*Cu/Tv*	−	Cu^2+^	8.73 ± 0.36 mg	+	2.6 × 10^4^
*H-Cu/Tv*					
*Ca-c*	+	Ca^2+^	9.98 ± 0.46 mg	−	−
*H-Ca-c*					
*Cu-c*	+	Cu^2+^	8.53 ± 0.55 mg	−	−
*H-Cu-c*					
*Ca/Tv-c*	+	Ca^2+^	10.11 ± 0.64 mg	+	1.1 × 10^5^
*H-Ca/Tv-c*					
*Cu/Tv-c*	+	Cu^2+^	8.87 ± 0.18 mg	+	3.2 × 10^4^
*H-Cu/Tv-c*					
*Tv* (*T. viride* spore suspension)	−	−	−	+	*1.4 × 10^6^ mL^−1^
*H-Tv*					
*C* (control)	−	−	−	−	−
*H-C*					

*Number of *T. viride* spores suspended in a saline solution (0.85%).

*Ca* – conventional/calcium-alginate microspheres; *H-Ca* – hydroponics/calcium-alginate microspheres; *Cu* – conventional/copper-alginate microspheres; *H-Cu* – hydroponics/copper-alginate microspheres; *Ca/Tv* – conventional/calcium-alginate microspheres with *T. viride* spores; *H-Ca/Tv* – hydroponics/calcium-alginate microspheres with *T. viride* spores; *Cu/Tv* – conventional/copper-alginate microspheres with *T. viride* spores; *H-Cu/Tv* – hydroponics/copper-alginate microspheres with *T. viride* spores; *Ca-c* – conventional/calcium-alginate microcapsules-chitosan coated; *H-Ca-c* – hydroponics/calcium-alginate microcapsules-chitosan coated; *Cu-c* – conventional/copper-alginate microcapsules-chitosan coated; *H-Cu-c* – hydroponics/copper-alginate microcapsules-chitosan coated; *Ca/Tv-c* – conventional/ calcium-alginate microcapsules with *T. viride* spores-chitosan coated; *H-Ca/Tv-c* – conventional/calcium-alginate microcapsules with *T. virid*e spores-chitosan coated; *Cu/Tv-c* – conventional/copper-alginate microcapsules with *T. viride* spores-chitosan coated; *H-Cu/Tv-c* – conventional/copper-alginate microcapsules with *T. viride* spores-chitosan coated; *Tv* – conventional/*T. viride* spore suspension in saline solution; *H-Tv* – hydroponics/*T. viride* spore suspension in saline solution; *C* – conventional/control; *H-C* – hydroponics/control. **Abbreviations in general: *Ca* or *Cu* is regarded to gelling cation which was used in the formation of alginate microbeads; *Tv* denotes the presence of *T. viride* spores in microparticles; large *H-* denotes hydroponics type of cultivation (*HC*) opposite of no denotation which is conventional cultivation (*CC*); small *-c* is regarded as alginate microbeads coated with chitosan.

**Figure 2:**
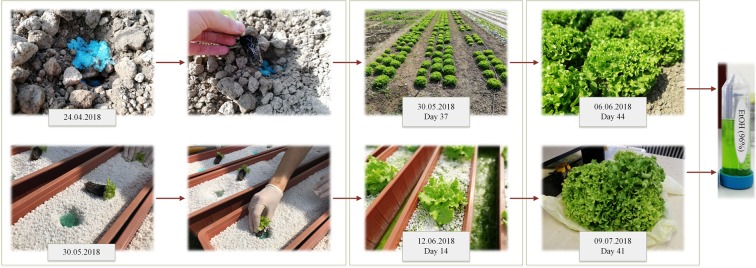
Schematic overview of the application and maturation of lettuces in two different types of cultivation (conventional vs. hydroponics).

The application was performed as weighing a 4 g of prepared microparticles and applying them directly near the root of a lettuce plant just before planting. Figure 2 shows the procedure and maturing time for two different types of cultivations. For the CC planting was performed at the Department of Vegetable Crops, Faculty of Agronomy, University of Zagreb during two years, 2017 and 2018. A single-factor assay with Melina lettuce was placed randomly in a block schedule in three repetitions (total of 540 seedlings). Field ground chemical analysis on the experimental area revealed: pH H_2_O – 7.5, nKCl – 6.86, Humus was 2.22%, N 0.2%, P_2_O_5_–41.1 and K_2_O – 25.5 as Al-mg/100 g. The distance between the seedlings in the row was 30 cm, while the spacing between the rows was also 30 cm. By planting the lettuces on these spaces, there was a complex of 11 plants × m^−2^ (18 plants per parcel). According to the available meteorological data from the Maksimir meteorological station, in the period from April to June, the average monthly air temperature ranged from 11.2–19.4 °C, which is optimal for outdoor lettuce growing. In all three months, 13 days were rainy and the average monthly precipitation was in the range from 61.5 mm in April to 96.8 mm in June.

Hydroponic cultivation of lettuce was carried out in 2018, in a protected area unsupplied with cooling system and energy curtains. The mono-factorial experiment was set up by the method of randomized complete block layout with four replications. Transplants of cv. ‘Melina’ with four developed leaves were planted on May 30 in growing pots filled with the inert substrate (perlite, 20 L per pot). During the planting, the microparticles were applied in the root-zone of transplants. Three transplants per pot were planted at a distance of 30 cm, and four pots per treatment were used. Growing pots were placed in pools tanked with a nutrient solution in composition recommended for leafy vegetables^35^. The nutrient solution was oxygen-enriched daily, but not supplemented or corrected. During the vegetation period, the abiotic parameters of the nutrient solution (temperature, pH and EC value, dissolved oxygen concentration) and air (minimum and maximum temperature and humidity) were monitored daily. Lettuce was harvested on July 9, and measurements carried out were: mass before and after primary finishing (g per rosette), diameter and height of rosette (cm), marketable yield (kg m^−2^).

### Plant measurements, moisture determination and preparation of extracts

Plants were harvested after 44 days in conventional and after 41 days in hydroponics cultivation and morphological parameters were taken (total head weight, diameter, height and marketable yield). Fresh lettuce was cut in quarters, washed with tap water to remove the soil/insects and then with distilled water. Washed lettuce was blotted with the paper towels to remove adherent water. Root tip was completely removed with the precise knife, and fresh lettuce quarters were chopped to the size of 4–6 mm in FOSS homogenizer 2094 (Hillerød, Denmark). Homogenized lettuce (4–6 mm) was subjected to dry matter/water content determination. Briefly, 5.00 g of fresh homogenized lettuce was subjected to the drying in PMB 202 Moisture Analyzer (Adam Equipment Company, United Kingdom) at 130 °C until completely dried.

Further, 30 g of freshly homogenized lettuce sample was taken in 100 mL of 96% ethanol for further extraction with a laboratory mixer and was homogenized for 30 s. Suspensions were filtered through the Whatman No. 4 filter and the volume of extracts were adjusted to 100 mL with the addition of solvent. Extraction was performed in triplicates and all of the chemical analyses following were performed in duplicates or triplicates (per one sample, 6–9 analyses were performed for each method, respectively).

### Chlorophylls analyses

The chlorophyll content was determined by extracting 1.0000 g of freshly homogenized lettuce with 25 mL of 80% (v/v) acetone by vortexing it for 2 minutes and filtering through the Whatman No. 4 filter paper. The final volume was set to 25 mL with the solvent. Absorbance was measured at 663 nm and 645 nm, and the chlorophylls content was calculated^36^. Results are expressed as mg of chlorophyll per dry weight of lettuce (µg/g d.w.).

### Determination of total polyphenolic content (TPC)

The modified Folin Ciocalteu’s method^37^ was used to determine total polyphenolic content (TPC). Ethanolic lettuce extract (0.1 mL) was mixed with 7.9 mL distilled water and 0.5 mL Folin Ciocalteu’s reagent (diluted with distilled water in 1:2 ratio) and 1.5 mL 20% sodium carbonate. The suspension was vortexed and left for 2 h to react. Absorbance was measured at 765 nm, and the data are expressed as gallic acid equivalents per g of dry weight (mg GAE/g d.w.).

### The total flavonoids (TF)

Total flavonoids content was determined by the modified spectrophotometric method^38^. Briefly, 1 mL of ethanolic lettuce extract was added in a 10 mL volumetric flask containing 4 mL of distilled water. Then, 300 μL of NaNO_2_ (0.5 g/L) solution was added. After 5 minutes, 300 μL of AlCl_3_ (1 g/L) solution was added and 6 minutes later, 2 mL of NaOH (1 mol/L) was added to the mixture. The final volume was set to 10 mL with the addition of distilled water. The solution was mixed and the absorbance was measured at 360 nm. Quercetin was selected as the standard and a seven-point standard calibration curve was plotted. The data are expressed as mg quercetin equivalents per dry weight of lettuce (mg QE/g d.w.).

### Antioxidant potential measurements – ABTS and DPPH methods

The antioxidant potential of the ethanolic lettuce extracts was determined with the 2,2-diphenyl-1-picrylhydrazyl (DPPH) and 2,2′-azino-bis (3-ethylbenzothiazoline-6-sulfonic acid) (ABTS) reagent, according to the known procedures^39,40^. The data obtained are expressed as μmol Trolox equivalents per g of dry weight (μmol TE/g d.w.).

### Statistical analysis

Statistical analysis was performed using Excel XLSTAT and IBM SPSS Statistics v22 software. One way ANOVA was performed and significant differences were observed based on the posthoc Tukey HSD test (p < 0.05). Principal component analysis (PCA) and agglomerative hierarchical clustering (AHC) were also performed. PCA type was set to Pearson (n) with filter factors a maximum number of 5. Bartlett’s test of sphericity was used to compare the correlation matrix with the matrix of zero correlations (significance level % was set to 5) The risk for the rejection of the null hypothesis H0, while it is true was <0.01%. Alpha was set to 0.05, and the p-value was <0.0001. The Keiser-Meyer-Olkin (KMO) measure of sampling adequacy was performed prior to the analysis. Further, Agglomerative hierarchical clustering (AHC) with Euclidean distance Dissimilarity and Agglomeration Ward’s method was performed.

## Results and Discussion

**Figure 3:**
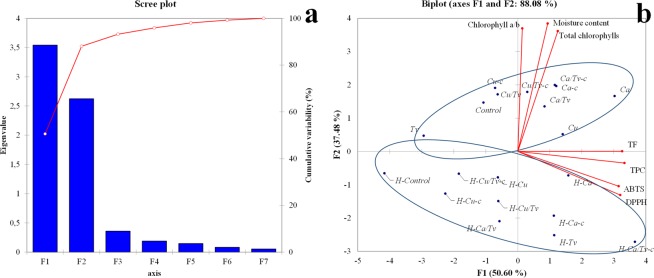
Principal component analysis Scree plot of the components (**a**) and plot of Factor 1 and Factor 2, with variables correlations (red lines). Abbreviations of investigated types of microparticles are presented in Table 1 and Principal component analysis summary statistics are in supplementary Table S1.

**Figure 4:**
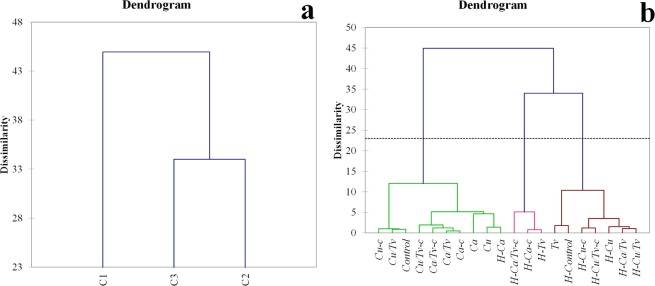
Agglomerative hierarchical clustering (AHC) dendrogram of dissimilarities and main cluster formations (**a**) and dendrogram formation of clusters after the microparticles treatments and analysis (**b**). Abbreviations of investigated types of microparticles are presented in Table 1.

Due to the simpler results presentation in Figs. 3 and 4 and in Tables 1 to 4, the prefix CC denoting conventional cultivation is excluded, and for hydroponic cultivation, the prefix is denoted as H instead of HC.

**Table 2:** Chlorophyll analysis of treated lettuce both in conventional (*CC*) and hydroponic (*HC*) cultivation with relative change respectively to the control.

Treatment	Chlorophyll *a* (µg/g d.w.)	*Relative change (%)	Chlorophyll *b* (µg/g d.w.)	*Relative change (%)	Total Chlorophylls (µg/g d.w.)	*Relative change (%)	Chlorophyll *a*:*b* ratio
***CC***							
*Ca*	1418.0 ± 119.7^a-c^	+45.0	530.7 ± 40.5^a,b^	+46.0	1948.7 ± 159.7^a-c^	+45.3	2.7
*Cu*	1201.9 ± 27.5	+22.9	492.9 ± 67.8^c^	+35.6	1694.8 ± 57.4^d^	+26.3	2.4
*Ca/Tv*	1247.2 ± 140.8	+27.5	478.6 ± 48.4	+31.7	1725.9 ± 134.1^e^	+28.7	2.6
*Cu/Tv*	1273.0 ± 105.3^d^	+30.2	479.7 ± 32.7	+32.0	1752.7 ± 135.2^f,g^	+30.7	2.7
*Ca-c*	1396.8 ± 138.5^e-g^	+42.8	527.1 ± 39.4^d^	+45.0	1923.9 ± 177.6^h-j^	+43.4	2.7
*Cu-c*	1098.0 ± 38.2^a,e^	+12.3	388.1 ± 49.1^a,d,e^	+6.7	1486.0 ± 72.5^a,h,k^	+10.8	2.8
*Ca/Tv-c*	1292.3 ± 108.5^h,i^	+32.2	470,2 ± 28.9	+29.3	1762.5 ± 137.4^l,m^	+31.4	2.7
*Cu/Tv-c*	1325.2 ± 69.3^j,k^	+35.5	528.5 ± 68.2^e,f^	+45.4	1853.7 ± 70.4^k,n,o^	+38.2	2.5
*Tv*	1010.9 ± 80.9^b,f,h,j^	+3.4	405.7 ± 32.6	+11.6	1416.6 ± 64.7^b,f,i,l,n^	+5.6	2.5
*Control*	977.9 ± 62.5^c,d,g,i,k^		363.6 ± 21.5^b-d,f^		1341.5 ± 83.6^c-e,g,j,m,o^		2.7
***HC***							
*H-Ca*	957.9 ± 53.0^a-i^	+76.2	391.8 ± 28.0^a-h^	+74.8	1349.7 ± 79.4^a-i^	+75.8	2.4
*H-Cu*	713.1 ± 79.2^a,j,k^	+31.2	296.5 ± 41.5^a,i,j^	+32.3	1009.6 ± 118.0^a,j,k^	+31.5	2.4
*H-Ca/Tv*	691.6 ± 30.3^b,l^	+27.2	307.8 ± 8.6^b,k,l^	+37.4	999.4 ± 26.4^b,l,m^	+30.2	2.2
*H-Cu/Tv*	696.4 ± 74.2^c,m^	+28.1	284.2 ± 29.8^c^	+26.8	980.6 ± 99.6^c,n^	+27.7	2.5
*H-Ca-c*	784.8 ± 75.1^d,n,o,p^	+44.4	337.1 ± 9.6^m-o,p^	+50.4	1121.9 ± 83.8^d,o-s^	+46.1	2.3
*H-Cu-c*	663.2 ± 98.9^e^	+22.0	274.9 ± 41.6^d^	+22.7	938.1 ± 140.3^e^	+22.2	2.4
*H-Ca/Tv-c*	593.5 ± 56.9^f,n^	+9.2	251.5 ± 23.6^e,m^	+12.2	845.0 ± 80.3^f,o^	+10.1	2.4
*H-Cu/Tv-c*	622.4 ± 49.6^g^	+14.5	252.8 ± 15.8^f,n^	+12.8	875.3 ± 65.2^g,p^	+14.0	2.5
*H-Tv*	501.5 ± 31.2^h,j,l,m,o^	−7.7	221.4 ± 22.9^g,i,k,o^	−1.2	723.0 ± 49.5^h,j,l,n,r^	−5.8	2.3
*H-Control*	543.6 ± 59.3^i,k,p^		224.1 ± 20.8^h,j,l,p^		767.7 ± 79.8^i,k,m,s^		2.4

*Relative change (%) with respect to the control treatment. Values superscripted with the same letter within a column are significantly different according to the posthoc Tukey HSD test (p < 0.05).

**Abbreviations: *Ca* or *Cu* is regarded to gelling cation which was used in the formation of alginate microbeads; *Tv* denotes the presence of *T. viride* spores in microparticles; large *H-* denotes hydroponics type of cultivation (*HC*) opposite of no denotation which is conventional cultivation (*CC*); small *-c* is regarded as alginate microbeads coated with chitosan.

**Table 3:** Share of bioactive compounds of treated lettuce both in conventional (*CC*) and hydroponic (*HC*) cultivation with relative change respectively to the control.

Treatment	Total flavonoids (mg QE/g d.w.)	*Relative change (%)	Total polyphenols (mg GAE/g d.w.)	*Relative change (%)
***CC***				
*Ca*	9.6 ± 0.2^a-d^	+15.2	4.4 ± 0.4^a,b^	+15.5
*Cu*	9.2 ± 0.3^e,f^	+10.8	4.2 ± 0.3^c^	+10.2
*Ca/Tv*	8.8 ± 0.5^g^	+6.5	4.2 ± 0.5^d^	+9.3
*Cu/Tv*	8.2 ± 0.3^a,h^	−0.8	3.7 ± 0.1^a^	−4.4
*Ca-c*	9.2 ± 0.4^i,j^	+11.3	4.4 ± 0.0^e^	+14.1
*Cu-c*	8.1 ± 0.2^b,e,i,k,l^	−3.1	3.8 ± 0.1	−1.0
*Ca/Tv-c*	9.3 ± 0.1^h,k,m^	+12.2	4.0 ± 0.3	+4.0
*Cu/Tv-c*	9.3 ± 0.8^l,n^	+11.5	4.1 ± 0.3	+6.5
*Tv*	7.3 ± 0.3^c,f,g,j,m,n^	−11.6	3.4 ± 0.2^b-e^	−11.1
*Control*	8.3 ± 0.2^d^		3.8 ± 0.1	
***HC***				
*H-Ca*	9.7 ± 0.2^ab^	+39.0	4.2 ± 0.4	+26.6
*H-Cu*	8.6 ± 0.3^c^	+23.1	4.0 ± 0.8	+19.9
*H-Ca/Tv*	8.4 ± 0.1^d^	+19.4	3.9 ± 0.2	+18.1
*H-Cu/Tv*	8.3 ± 0.7^e^	+19.1	3.7 ± 0.6	+11.2
*H-Ca-c*	8.9 ± 0.9^f^	+26.5	4.1 ± 0.2	+22.2
*H-Cu-c*	8.0 ± 0.1^ag^	+13.9	3.5 ± 0.1^a^	+5.2
*H-Ca/Tv-c*	10.0 ± 0.7^d,e,g,h^	+42.2	4.9 ± 0.6^a-c^	+47.6
*H-Cu/Tv-c*	8.9 ± 0.1^i^	+26.4	3.4 ± 0.6^b^	+1.8
*H-Tv*	9.0 ± 0.8^j^	+28.6	4.3 ± 0.2	+28.1
*H-Control*	7.0 ± 0.9^b,c,f,h-j^		3.3 ± 0.1^c^	

*Relative change (%) with respect to the control treatment. Values superscripted with the same letter within a column are significantly different according to the posthoc Tukey HSD test (p < 0.05).

**Abbreviations: *Ca* or *Cu* is regarded to gelling cation which was used in the formation of alginate microbeads; *Tv* denotes the presence of *T. viride* spores in microparticles; large *H-* denotes hydroponics type of cultivation (*HC*) opposite of no denotation which is conventional cultivation (*CC*); small *-c* is regarded as alginate microbeads coated with chitosan.

**Table 4:** Antioxidant activity of treated lettuce both in conventional (*CC*) and hydroponic (*HC*) cultivation with relative change respectively to the control.

Treatment	ABTS (µmol TE/g d.w.)	*Relative change (%)	DPPH (µmol TE/g d.w.)	*Relative change (%)
***CC***				
*Ca*	21.5 ± 1.0^a-g^	+29.7	17.1 ± 1.0^a-d^	+32.4
*Cu*	18.3 ± 1.3^h^	+10.3	16.5 ± 0.4^e-g^	+27.9
*Ca/Tv*	17.6 ± 0.4^a^	+6.3	15.7 ± 0.7^h,i^	+21.8
*Cu/Tv*	17.7 ± 1.7^b^	+6.7	14.0 ± 0.7^a,e^	+8.4
*Ca-c*	17.0 ± 0.7^c^	+2.6	15.3 ± 0.6^j^	+18.2
*Cu-c*	16.9 ± 1.6^d^	+1.8	14.8 ± 0.7	+14.2
*Ca/Tv-c*	18.8 ± 1.8^i^	+13.7	15.4 ± 1.8^k,l^	+19.0
*Cu/Tv-c*	15.7 ± 1.5^e^	−5.0	14.3 ± 0.8^b^	+10.4
*Tv*	14.6 ± 0.1^f,h,i^	−12.0	12.6 ± 0.7^c,f,h,j,k^	−2.4
*Control*	16.6 ± 0.8^g^		12.9 ± 0.6^d,g,i,l^	
***HC***				
*H-Ca*	18.5 ± 1.7^a^	+37.7	17.0 ± 2.4^a^	+48.5
*H-Cu*	17.3 ± 1.1	+28.3	13.8 ± 0.2^b^	+20.8
*H-Ca/Tv*	18.0 ± 0.7	+33.6	15.1 ± 0.8	+31.8
*H-Cu/Tv*	18.1 ± 0.1	+34.3	16.1 ± 2.0	+40.6
*H-Ca-c*	19.8 ± 1.9^b^	+47.3	17.8 ± 0.4^c,d^	+56.1
*H-Cu-c*	16.7 ± 0.1^c^	+24.3	12.6 ± 0.4^c,e,f^	+10.7
*H-Ca/Tv-c*	22.1 ± 2.3^c,d,e^	+63.9	19.3 ± 1.8^b,e,g,h^	+69.0
*H-Cu/Tv-c*	15.8 ± 2.7^d^	+17.7	13.1 ± 0.3^g^	+14.8
*H-Tv*	19.0 ± 1.7^f^	+41.3	17.9 ± 1.8^f,i^	+56.6
*H-Control*	13.4 ± 2.3^a,b,e,f^		11.4 ± 1.2^a,d,h,i^	

*Relative change (%) with respect to the control treatment. Values superscripted with the same letter within a column are significantly different according to the posthoc Tukey HSD test (p < 0.05).

**Abbreviations: *Ca* or *Cu* is regarded to gelling cation which was used in the formation of alginate microbeads; *Tv* denotes the presence of *T. viride* spores in microparticles; large *H-* denotes hydroponics type of cultivation (*HC*) opposite of no denotation which is conventional cultivation (*CC*); small *-c* is regarded as alginate microbeads coated with chitosan.

With regards to the morphological parameters (total head weight, diameter, height and marketable yield) there were no statistically significant differences found after the treatment with microparticles or *T. viride* spores suspended in saline solution when compared to the control or the treatments according to the posthoc Tukey HSD test. There was no significant influence (p < 0.05) of treatments with microparticles on the moisture content of the lettuces either. Even though somewhat lower percentage of water content was observed in the samples cultivated in hydroponics (lowest in *H-Cu-c* with 92.1% and highest for *H-Cu* samples with 92.9%) compared to the conventional (lowest for the *Tv* treatment with 92.9% and highest for the *Cu/Tv-c* treated sample with 94.3%), the results are in accordance with the literature where about 93% of water content was found in Batavia species^41^. No statistically significant changes reveal there was no effect of the treatments on the lettuce moisture content or morphology.

### Changes in chlorophylls content

When comparing the cultivation type of lettuce, as conventional cultivation (CC) and hydroponics (HC), samples cultivated conventionally had a significantly higher content of chlorophylls (compared to the equal treatments in HC) which can be explained by the fact that hydroponically grown lettuces were in the greenhouse (Table 2), since more light directly affects photosynthesis, the production of more chlorophylls outside the greenhouse is expected. Light and heath conditions are the main factors affecting the plant physiology with the direct effect on the photosynthesis mechanisms. Exposure to the light determinates the quantity and quality of the energy available to the photosynthesis machinery to conduct its normal metabolic activities^42^.

When comparing CC and HC with different microparticles or *Tv* treatments, a high correlation can be observed (*r*_*TotalChlorophylls*_ = 0.75) which verifies the repeatability and feasibility of this experiment (since the same experiment is replicated in two different types of cultivation). Interestingly, statistically significant and the most pronounced effect on the lettuce treatment (in terms of chlorophylls) had microparticles containing Ca^2+^ ions, without the *Tv* presence. Additionally, calcium-based microcapsules had less effect than microspheres on the lettuces chlorophylls production due to the slower release of the ions and the lower cations availability to a plant. The least feasible effect on the production of the chlorophylls in the lettuces had the treatments with only a suspension of *Tv* (this is with the respect to the appropriate controls). This may be explained by the lower survivability of the non-encapsulated *Tv*, as well as the need for this fungus to uptake some of the nutrients from the surrounding media (ground) in order to survive (thus, the same or even negative impact on the plant). Respectively, the treatments with microparticles containing both chemical agents and *Tv* spores had a similar or lower effect on the production of chlorophylls in the lettuces compared to the same microparticles without the *Tv*. Similarly, we have previously found higher content of chlorophylls in the leaves of the *Vitis vinifera* L. plants treated with Ca^2+^ loaded microspheres as well as with the combination of Ca^2+^/Mg^2+^ loaded microspheres^4^. This can be ascribed to the electrostatic binding of the cations to negatively charged *Tv* spores which we confirmed in our previous research^30–32^.

Furthermore, the relative change in the total chlorophylls content was up to 45.3% for the CC and 75.8% for the HC treatments with Ca^2+^ microparticles, suggesting that the same microparticles can stimulate the production of more chlorophylls even with the less available light. The availability to uptake calcium ions is due to the fact that in the microparticles, calcium remains in the Ca^2+^ form, which is passively uptaken by the plant through the root system^43^. Our results are in accordance with the research with^15^ where they observed higher values of chlorophyll *a*, *b* and total chlorophylls in chickpea leaves supplemented with Ca^2+^. Even more, the pronounced effect was observed in the cadmium stressed plants where the application of Ca^2+^ improved synthesis and protection of the photosynthetic pigments. Furthermore, Ca^2+^ serves as a secondary messenger for cytokinin action in improving the synthesis of chlorophylls^44^.

Ca^2+^ ions are free to enter roots passively but the transfer to the shoots is limited by a metabolic barrier. Some of the metabolic inhibitors can stop the long-distance transport of Ca^2+^ to the shoots but cannot inhibit the uptake of the roots. Since Mg^2+^ and many other trace metals result in phytotoxicity when Ca^2+^ share is low, evidently the supply and availability of Ca^2+^ ions are important. When Ca^2+^ concentrations in the plants are low, phytotoxicities from trace metals are very common and even Mg^2+^ ions become extremely phytotoxic^43,45^. Since microparticles containing the Ca^2+^ ions are present throughout the period of the plant maturation, this has proved to be an efficient delivery system with a direct influence on the chlorophylls and the photosynthesis machine. Ca^2+^ ions control and modify the uptake of available Mg^2+^ and nitrogen, which are both important components of the chlorophyll structure^46^. Moreover, using the calcium-based microparticles, it is possible to eliminate the competition between magnesium and calcium ions thus increasing the availability of Mg^2+^ for plant uptake and at the same time preventing the occurrence of negative metabolic changes of the plant leaves. Accordingly, by increasing the number of magnesium ions to the plant, synthesis of more chlorophylls can be achieved in the leaves of the plant and thus decrease in the level of abiotic and biotic stress of the plant can occur.

Even though the chlorophyll a:b ratio was somewhat higher in the conventionally cultivated lettuces, there were no statistically significant differences found between the cultivation types under the different light conditions (the chlorophyll a:b ratio behaved similarly). The slightly higher chlorophyll a:b ratio in the conventional cultivation (average for CC 2.63 vs. average for HC 2.38) is in correlation with the literature, where an increase of the ratio is correlated with the available light. The ratio is physiologically flexible, and it is least influenced by the soil status or the water availability and is dissociated from patterns imposed by changes in leaf density^47^.

### Total polyphenolic content (TPC) and total flavonoids (TF)

When comparing the “basic” metabolism, which comprises of anabolic/catabolic processes which are required for the cell maintenance and proliferation, PSM refers to the compounds that are present in the specialized cells which are not directly essential for the basic metabolism but are required for plant survival in the environment. PSM was considered simply as waste products of primary metabolism which accumulates in the plant cells because of the absence of an efficient excretion^48–50^, but that idea is not valid. Plant secondary metabolites (*i.e*. polyphenols) act as a defense (against herbivores, microbes, viruses or competing plants) and also as signal compounds (to attract pollinating or seed-dispersing animals) and they offer protection to the plants against ultraviolet radiation and oxidation processes^51,52^. These metabolites can, therefore, be acknowledged as adaptive characters that have been subjected to natural selection during the evolution. Compared to the animals, plants cannot escape from their biotic and abiotic stressors by being linked to the ground via their root system, thus because they are static the protection must be commenced in another way. Phytochemicals that they produce are to deter or kill pests and pathogens and this represents one point of self-protection^5^. Since polyphenols and flavonoids have high antioxidant activity and are electron-donating compounds^53^, they can scavenge reactive oxygen species. Polyphenolic compounds are generally synthesized through the signaling processes^54,55^. The role of Ca^2+^ in polyphenolic metabolism has been described by various authors and starting with a paper by^56^ demonstrated the direct role of Ca^2+^ in the synthesis of polyphenolic compounds. The application of Ca^2+^ increased phenylalanine ammonia-lyase activity, which ended with the accumulation of polyphenols and thus increasing the resistance to the infection by the specific fungus. Furthermore, Ca^2+^ indirectly activates peroxidase, as this cation induces the cross-linking of polygalacturonan chains into a structure that can be recognized by isoperoxidase^57^.

Different cultivation types did not significantly affect the accumulation of TPC in lettuces but the higher relative change was observed in the treatment of HC lettuces, respectively. Due to the fact that hydroponics is a more controlled environment (compared to conventional cultivation) the influence of the treatments was more clear. TPC of the treated samples was higher in *H-Ca/Tv-c* treated lettuces with the significant influence and the relative change of 47.6% compared to the control. From Table 3 it can be observed that treatments with Ca^2+^ based microparticles had a somewhat stronger effect on the synthesis of polyphenolic compounds compared to Cu^2+^ based microparticles.

The significant effect on the TF accumulation with the microparticles treatments can be observed on the samples cultivated in hydroponics, where microparticles based on calcium-alginate (loaded with Ca^2+^ ions with or without *T. viride* spores) achieved an increase from 19.4% (*H-Ca/Tv*) to 42.2% (*H-Ca/Tv-c*). In this case, it seems that chitosan coating prevents the burst release of the Ca^2+^ (which is in accordance with our *in vitro* tests^33^) so the uptake to the root was slower (rate controlled) than those lettuces treated with microspheres. Due to the preparation conditions (to achieve the necessary solubility of the chitosan) microcapsules are more acidic. There is some literature dealing with the influence of the lower pH on the production of TF, where there was found a high correlation between the low pH and higher TF content in leaves. By this, the pH of the surrounding media strongly affects the accumulation of PSM which can lead to a significant enhancement in the productivity of flavonoids^58^. In both types of cultivation (CC and HC) calcium-alginate microparticles (*Ca* and *H-Ca*) treatment had the most pronounced effect and statistically significantly influenced, compared to the respective controls with the relative change of 15.2% for the CC and 39.0% for the HC. In general, somewhat lower changes were observed in CC compared to the HC, but this can be explained by the lower values of TF for the control in HC (7.0 mg QE/g d.w.) compared to the control from the CC (8.3 mg QE/g d.w.). Recently was proved that flavonoids in sprouts were accumulated more under light irradiation than under dark^59^. Therefore, CC is evidently more favorable for the production of lettuces with higher TF. Results of the influence on TPC are in correlation with regards to the TF content, but with no statistically significant changes against the control, except in the case of HC for the *H-Ca/Tv-c* treatment (relative change – 47.6%).

*Tv* spore suspension in HC had a significantly higher influence on the synthesis of TPC and TF with the high antioxidant activity of lettuces. Due to the fact that in CC *Tv* suspension was injected into the soil, which is already contaminated with other microorganisms, non-encapsulated *Tv* had no effect or somewhat negative effect on the production of PSM. Also, non-encapsulated *Tv* had no significant effect on the production of chlorophylls, in both types of cultivation, but significantly increased TPC and TF in HC. This can be explained because in hydroponics, when planting, the cleaner environment was achieved, with no competition for *T. viride*, thus it could thrive. The results of^60^ indicated that some *Trichoderma* sp. were able to increase the total amount of polyphenols and antioxidant activity in the grapes (*Vitis vinifera* L.). In our experiment, the treatment with non-encapsulated *T. viride* achieved significant influence, with the relative change of 28.1% compared to the control^4^.

### Influence of the treatments on the antioxidant activity (AA)

Ahmad *et al*.^15^ show that supplementation with Ca^2+^ boosts antioxidant activity in plants. Again, as shown in Table 4, in CC and HC, Ca^2+^ microparticles had a significant effect on the antioxidant activity of lettuce. In general, Cu^2+^ based microparticles, with or without *T. viride*, had a lower effect on AA compared to the Ca^2+^ based, but this can be explained by lowering the activity of antioxidant enzymes. In higher concentrations, Cu^2+^ can indirectly act as pro-oxidants. This may increase free radical-mediated lipid peroxidation in plants. Since moderate-high dosages of Cu^2+^ may activate zymogen or trigger the defensive system by ROS, antioxidant enzymes activity decreases, thus limiting the elimination of ROS. Alongside, MDA concentration can get higher in plants exposed to moderate-high Cu^2+^ concentrations thus indirectly produce superoxide radicals, resulting in increased lipid peroxidative products and oxidative stress^61,62^. This might be partly the explanation of why Cu^2+^ based microparticles had less influence on the lettuces AA, even though compared to the control the AA was higher.

Accordingly, to the results of TPC and TF, *Tv* had a significant influence on the AA of lettuces grown in HC, whereas there was no significant effect on lettuces grown in CC. The results are in correlation, and with the increased TPC and TF, antioxidant activity is higher, suggesting that *T. viride* in the right conditions plays a significant role in the synthesis of polyphenols, probably indirectly by enhancing the nutrient availability and by inducing the plant resistance via activating or priming induced systemic resistance mechanism^60^.

### Pearson correlations, principal component analysis (PCA) and agglomerative hierarchical clustering (AHC) analyses

High correlations with regards to the comparison of CC and HC for all of the methods with the highest correlation between the TF in CC and HC lettuces ($${r}_{TF(CC/HC)}=0.87)$$ were found. The high correlation between the antioxidant activity methods was also observed ($${r}_{ABTS/DPPH)}=0.92)$$. Furthermore, when observing the methods, high correlations were found for TPC, TF and antioxidant activity (*r* > 0.80) and chlorophylls content (*r* > 0.99). No correlation was found in the relationship between chlorophylls content and TPC/TF/AA.

PCA was used to indicate multivariate dependence between the selected variables. The weighting of the results was made for the moisture content, chlorophyll a:b, total chlorophylls, TPC, TF, ABTS, and DPPH. For Bartlett’s Sphericity test, the risk for the rejection of the null hypothesis H0 while it was true was <0.01%. Alpha was set to 0.05, and the *p*-value was <0.0001. The Kaiser-Meyer-Olkin (KMO) measure of sampling adequacy gave a value of 0.734. High factor loading scores mean a tighter association with the same principal component (Table S1). PCA revealed the two significant components which explain altogether 88.08% of the total variance between the studied variables (Fig. 3a). Factor 1 (F1) describes 50.60% of the total variance and was tightly associated with the TPC, TF, ABTS, and DPPH whereas Factor 2 (F2) was associated with moisture content, chlorophyll a:b and total chlorophylls content. Figure 3b presents the distribution of the data for F1 and F2 and the correlation between the measured variables. There was no correlation found between F1 (TPC, TF, ABTS, DPPH) and F2 (moisture content, chlorophyll a:b, and total chlorophylls).

The dendrogram (Fig. 4a) was obtained based on the same variables as PCA, and it can be seen that three main clusters were formed (green, pink and red). HC lettuce treatments were separated in 2 closely related clusters (pink and red) where *Tv* from CC was also involved. Based on the obtained results (Fig. 4b), *Tv* from had the least influence on the CC treatments compared to the other treatments and was similar to the control of the latter, but because of the lower values of investigated PSM, it clustered with the control of HC. In general, Cu^2+^ based microparticles in CC are closely related to the control. In the first cluster (green) it can be observed that *H-Ca* treatment is more similar to the values of the CC type, and this is explained by the fact that Ca^2+^ microparticles in HC had the significant and highest influence on the plant secondary metabolites. Biplot (Fig. 3b) reveals similar results, where CC lettuces were divided from HC.

*Ca* (top right quadrant) and *H-Ca/Tv-c* (bottom right quadrant) treatments can be seen as extremes on the right side of the biplot (Fig. 3b), and *Tv* alongside *H-control* on the left side. In both cases, control is significantly distanced from the *Ca* and *H-Ca/Tv-c* treatments, signifying the influence of these treatments on the lettuces development of PSM. Cu^2+^ microparticles formulations are in general grouped together (respectively to the appropriate type of cultivation). It seems that *Ca/Tv-c*, *Ca-c* and *Ca/Tv* had a somewhat similar and significant effect on the synthesis of PSM, and *Cu/Tv-c*, *Cu/Tv* and *Cu-c* are more closely related to the control. This is in correlation to the HC where *H-Cu/Tv*, *H-Cu-c*, *H-Cu* and *H-Cu/Tv* are closer to the control, compared to their Ca^2+^ counterparts microparticles, *H-Ca/Tv*, *H-Ca*, *H-Ca-c* and *H-Ca/Tv-c*. The only exception in two cases of cultivation types is *Tv*, where *Tv* in CC had no significant influence, but *H-Tv* in HC had a significant influence on the TPC, TF, ABTS and DPPH and is closely related to the extreme in HC (*H-Ca/Tv-c*). PCA and AHC revealed the significant influence of some treatments (*i.e*. Ca^2+^ based microparticles) on lettuces in both conventional and hydroponic cultivation.

## Conclusions

Delivery of biological and chemical agents by means of encapsulation presents an innovative approach to stimulate the production of PSM. This way, it is possible to fortify the plants’ defense system against pests and increase the resistance to harsh environmental conditions.

Results revealed that microparticles treatments significantly stimulated the synthesis of PSM without a significant impact on the lettuce morphology and moisture content. The controlled release was achieved and the plant can uptake ions passively through the root system during the whole period of maturation. Evidently, non-encapsulated *T. viride* spores had significantly less influence on the lettuces compared to the encapsulated ones. Ca^2+^ based microparticles had a higher effect on the synthesis of PSM compared to their counterparts Cu^2+^ based microparticles, even though significant changes occurred in all treatment types, respectively.

The final product (microparticles) is commercially very affordable and further is necessary only to upscale the production process. Bioencapsulation proved to be an efficient way to deliver both chemical and biological agents for plant nutrition/protection and the production of functional foods. Our method may be applied in the production of plants (in this case lettuce) with improved nutritional quality and as a potential dietary source of natural phenolic antioxidants.

## Supplementary information

Supplementary material.
