# Supplementary material for: The enhancement of plant secondary metabolites content in Lactuca sativa L. by encapsulated bioactive agents
Source: Sci Rep. 2020 Feb 28;10:3737. doi: 10.1038/s41598-020-60690-3 (PMC7048752; doi:10.1038/s41598-020-60690-3)
Supplement: Supplementary file 1 — Supplementary material. [file 41598_2020_60690_MOESM1_ESM.docx]

**Supplementary material**

**The enhancement of plant secondary metabolites content in *Lactuca sativa* L. by encapsulated bioactive agents**

Slaven Jurić^1^, Katarina Sopko Stracenski^1^, Żaneta Król-Kilińska^4^, Ivanka Žutić^3^, Sanja Fabek Uher^3^, Edyta Đermić^2^, Snježana Topolovec-Pintarić^2^, Marko Vinceković^1*^

University of Zagreb, Faculty of Agriculture, Department of Chemistry^1^, Department of Plant Pathology^2^, Department of Vegetable Crops^3^ and Wrocław University of Environmental and Life Sciences, Faculty of Biotechnology and Food Sciences, Department of Functional Food Products Development^4^

**Corresponding author:** Marko Vinceković, University of Zagreb, Faculty of Agriculture, Department of Chemistry, mvincekovic@agr.hr

*Microsphere preparation*

Two types of microparticles (microspheres and microcapsules) were prepared as described before^1-5^. Microspheres (Fig. 1) consisted of the gelling cation (Ca^2+^ or Cu^2+^) with or without *Trichoderma viride – Tv*. They were prepared by dripping sodium alginate (1.5%) or a mixture of sodium alginate (1.5%) and *T. viride* spores into calcium chloride solution (1%) or copper(II) sulfate pentahydrate solution (1%). The production of microparticles was achieved with Encapsulator (Büchi-B390, BÜCHI Labortechnik AG, Switzerland) with the flow rate of carrier solution of 30 to 40 mL min^–1^ (determined by using encapsulator nozzle size of 1000 μm) at the vibration frequency of 40 Hz and the pressure of 0.3 bar. Microparticles were formed in the cross-linking solution (containing either Ca^2+^ or Cu^2+^) under mechanical stirring, then washed several times with distilled water and filtered through Büchner funnel. Microcapsules (Fig. 1) were prepared by dispersing microspheres in chitosan solution (0.5% chitosan in 1.0% CH_3_COOH) under constant stirring for 30 minutes. Obtained microcapsules were filtered, washed with distilled water and saline buffer. Microparticles were used the next day on the field with control as non-treated lettuces. Alginate-based microspheres containing either only chemical (Ca^2+^ or Cu^2+^) or both chemical and biological agents (*Trichoderma viride* spores), and the above-mentioned combinations but with chitosan coating are labeled and treatment samples are listed in Table 1. A total of 8 types of microparticles were produced (4 types of microspheres (no chitosan coating) and 4 types of microcapsules (chitosan coating)). Abbreviations of microparticles and cultivation type (conventional or hydroponics) are listed below:

*Ca* – conventional/calcium-alginate microspheres;

*H-Ca* – hydroponics/calcium-alginate microspheres;

*Cu* – conventional/copper-alginate microspheres;

*H-Cu* – hydroponics/copper-alginate microspheres;

*Ca/Tv* – conventional/calcium-alginate microspheres with *T. viride* spores;

*H-Ca/Tv* – hydroponics/calcium-alginate microspheres with *T. viride* spores;

*Cu/Tv* – conventional/copper-alginate microspheres with *T. viride* spores;

*H-Cu/Tv* – hydroponics/copper-alginate microspheres with *T. viride* spores;

*Ca-c* – conventional/calcium-alginate microcapsules-chitosan coated;

*H-Ca-c* – hydroponics/calcium-alginate microcapsules-chitosan coated;

*Cu-c* – conventional/copper-alginate microcapsules-chitosan coated;

*H-Cu-c* – hydroponics/copper-alginate microcapsules-chitosan coated;

*Ca/Tv-c* – conventional/ calcium-alginate microcapsules with *T. viride* spores-chitosan coated;

*H-Ca/Tv-c* – conventional/calcium-alginate microcapsules with *T. viride* spores-chitosan coated;

*Cu/Tv-c* – conventional/copper-alginate microcapsules with *T. viride* spores-chitosan coated;

*H-Cu/Tv-c* – conventional/copper-alginate microcapsules with *T. viride* spores-chitosan coated;

*Tv* – conventional/*T. viride* spore suspension in saline solution;

*H-Tv* – hydroponics/*T. viride* spore suspension in saline solution;

*C* – conventional/control;

*H-C* – hydroponics/control.

**Abbreviations in general: Ca or Cu is regarded to gelling cation which was used in the formation of alginate microbeads; *Tv* denotes the presence of *T. viride* spores in microparticles; large *H-* denotes hydroponics type of cultivation (HC) opposite of no denotation which is conventional cultivation (CC); small *-c* is regarded as alginate microbeads coated with chitosan.

*Loading capacity*

Loading capacity is defined as the content of loaded calcium (Ca^2+^) or copper (Cu^2+^) cations per gram of microparticles. The cations content was determined by dissolving 10 mg of dry microcapsules in 5 ml of a mixture of 0.2 M NaHCO_3_ and 0.06 M Na_3_C_6_H_5_O_7_ x 2H_2_O at pH 8.2^6^. The resulting solution was filtered and the concentration of calcium ions in the filtrate was determined with the Arsenazo III method^7^ by UV-VIS spectrophotometer at 650 nm and for copper ions at 795 nm (Shimadzu, UV-1700, Japan). The number of *T. viride* spores was determined in the filtrate at 550 nm by the method of Waghunde et al.^8^ at 550 nm (Shimadzu, UV-1700, Japan). The number of *T. viride* spores suspended in a saline solution (0.85%) was determined directly from the solution at 550 nm. Chemical agent share and number of *Trichoderma viride* spores are presented as per 4 g of microparticles in Table 1. The measurements were replicated six times and the results are expressed as mean values with standard deviation.


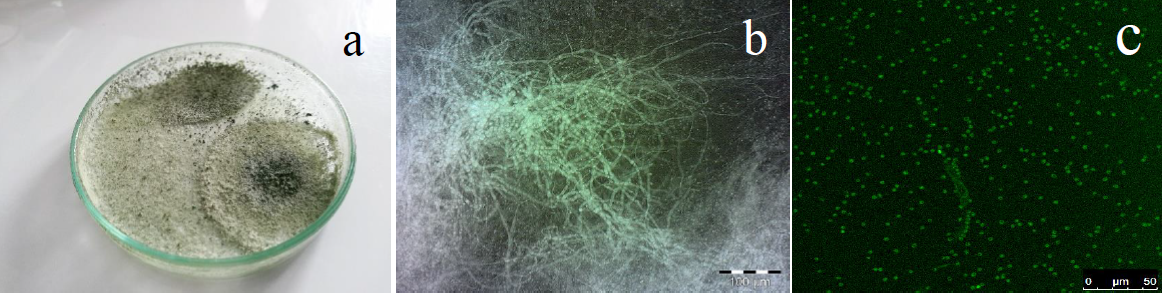


**Figure S1.** (a) Macrophotograph of growing *T. viride* in a Petri dish, and microphotographs of *T. viride* (b) mycelium and (c) spore suspension stained with Rhodamine 123 fluorescent dye (CLSM) (bars are indicated).

**Table S1.** Principal component analysis summary statistics.

|  | **Factor** | **Factor loading** | | **Eigenvector** | | **Contribution of variables (%)** | | **Correlations between variables and factors:** | |
| --- | --- | --- | --- | --- | --- | --- | --- | --- | --- |
|  |  | F1 | F2 | F1 | F2 | F1 | F2 | F1 | F2 |
| Measures | *Moisture content* | 0.264 | 0.934 | 0.140 | 0.577 | 1.971 | 33.248 | 0.264 | 0.934 |
|  | *Total chlorophylls* | 0.353 | 0.879 | 0.188 | 0.543 | 3.518 | 29.431 | 0.353 | 0.879 |
|  | *Chlorophyll a:b* | 0.039 | 0.898 | 0.021 | 0.554 | 0.044 | 30.735 | 0.039 | 0.898 |
|  | *TF* | 0.921 | 0.001 | 0.489 | 0.001 | 23.946 | 0.000 | 0.921 | 0.001 |
|  | *TPC* | 0.942 | -0.085 | 0.500 | -0.053 | 25.037 | 0.272 | 0.942 | -0.085 |
|  | *ABTS* | 0.890 | -0.254 | 0.473 | -0.157 | 22.387 | 2.454 | 0.890 | -0.254 |
|  | *DPPH* | 0.904 | -0.319 | 0.481 | -0.196 | 23.096 | 3.858 | 0.904 | -0.318 |
| Eigenvalues | *Eigenvalue* | 3.542 | 2.623 |  |  |  |  |  |  |
|  | *Variability (%)* | 50.601 | 37.478 |  |  |  |  |  |  |
|  | *Cumulative (%)* | 50.601 | 88.078 |  |  |  |  |  |  |

1. Vinceković, M. *et al.* Encapsulation of Biological and Chemical Agents for Plant Nutrition and Protection: Chitosan/Alginate Microcapsules Loaded with Copper Cations and *Trichoderma viride*. *J. Agric. Food Chem.* **64**(*43*), 8073-8083 (2016). <https://doi.org/10.1021/acs.jafc.6b02879>
2. Vinceković, M., Jurić S., Đermić, E. & Topolovec-Pintarić, S. Kinetics and Mechanisms of Chemical and Biological Agents Release from Biopolymeric Microcapsules. *J. Agri. Food Chem.* **65**(*44*), 9608-9617 (2017). <https://doi.org/10.1021/acs.jafc.7b04075>
3. Vinceković, M. *et al.* Release of *Trichoderma viride* Spores from Microcapsules Simultaneously Loaded with Chemical and Biological Agents. *Agric. Conspec. Sci.* **82**(4), 395-401 (2017). <https://hrcak.srce.hr/193536>
4. Jurić, S., Đermić, E., Topolovec-Pintarić, S., Bedek, M. & Vinceković, M. Physicochemical properties and release characteristics of calcium alginate microspheres loaded with *Trichoderma viride* spores. *J. Integr. Agr.* **18**, 3-16 (2019). doi:10.1016/S2095-3119(19)62634-1
5. Jurić, S., Šegota, S. & Vinceković, M. Influence of surface morphology and structure of alginate microparticles on the bioactive agents release behavior. *Carbohydr. Polym.* **218**, 234-242 (2019). <https://doi.org/10.1016/j.carbpol.2019.04.096>
6. Li, X.Y. *et al.* Chitosan-alginate microcapsules for oral delivery of egg yolk immunoglobulin (IgY). *J. Agric. Food Chem.*, **55**(*8*), 2911-2917 (2007). doi: 10.1021/jf062900q
7. Janssen, J. W. & Helbing, A. R. Arsenazo III: an improvement of the routine calcium determination in serum. *Eur. J. Clin. Chem. Clin. Biochem.* **29**(*3*), 197-201 (1991). PMID: 2070016
8. Waghunde, R. R., Priya, J., Naik, B. M., Solanky, K. U., Sabalpara, A. N. Optical density – A tool for the estimation of spore count of *Trichoderma Viride*. *J. Biopesticides.* **3**, 624–626 (2010).
